# Exploring the Therapeutic Value of Some Vegetative Parts of *Rubus* and *Prunus*: A Literature Review on Bioactive Profiles and Their Pharmaceutical and Cosmetic Interest

**DOI:** 10.3390/molecules30153144

**Published:** 2025-07-26

**Authors:** Andreea Georgiana Roșcan, Irina-Loredana Ifrim, Oana-Irina Patriciu, Adriana-Luminița Fînaru

**Affiliations:** 1 Doctoral School in Environmental Engineering, “Vasile Alecsandri” University of Bacau, 157 Marasesti Str., 600115 Bacau, Romania; anroscan99@yahoo.ro; 2 Department of Chemical and Food Engineering, “Vasile Alecsandri” University of Bacau, 157 Marasesti Str., 600115 Bacau, Romania; oana.patriciu@ub.ro

**Keywords:** plant waste, extraction, phytochemicals, biological activity, synergistic effect

## Abstract

The resulting plant waste from *R. idaeus*, *P. serotina*, *P. avium*, and *P. cerasus* exhibits a complex chemical composition, depending on the variety from which it originates, with applications in multiple fields such as the food, pharmaceutical or dermato-cosmetic industry due to the presence of phytochemical compounds such as flavonoids, flavonols, tannins, cyanogenic glycosides, vitamins, aldehyde, and phenolic acids. The aim of this review was to summarize and analyze the most recent and significant data from literature on the importance of plant waste resulting from the pruning process of trees and shrubs, in the context of applying circular economy principles, with a focus on the pharmacological importance (antimicrobial, antioxidant, anti-inflammatory, anticoagulant, antiviral, and antitumoral) of some bioactive compounds identified in these species. Their applicability in various industries is closely linked to both the bioavailability of the final products and the study of their toxicity. The literature indicates that the isolation of these compounds can be carried out using conventional or modern methods, the last ones being favored due to the increased efficiency of the processes, as well as from the perspective of environmental protection. This review increases the attention and perspective of using plant waste as a linked source of pharmaceutical and dermato-cosmetic agents.

## 1. Introduction

The concept of the circular economy can be effectively applied in the management of plant waste by valorizing by-products in alternative industrial processes [1]. Specialized literature highlights that the plant waste resulting from orchard maintenance work can be utilized by mulching the land, thus supporting the principle of the circular economy, and, in addition to the benefit of ecological fertilization, financial sustainability is also added [2]. Although current technologies for their valorization are promising, their large-scale applicability is still limited and requires further studies on the technical-economic viability of the methods [3].

Additionally, there are other methods for valorizing these wastes, but not all of them have a minimally invasive or even positive impact on the environment. For example, the use of woody biomass as an energy source has a negative ecological impact on the environment, highlighting the need to research more ecological and efficient alternatives for its valorization [4].

Gas emissions and fine particles resulting from combustion contribute to global warming, but human health is also impacted by air pollution [2,5]. A study from the USA highlighted that emissions from the bioenergy sector contribute a maximum of 17% to total emissions from all energy [6].

A viable method of valorization on cherry and sour cherry woody biomass resulting from orchard renewal is to use the wood to make barrels that are used in the food industry for aging distillates, wines, or vinegar [7]. In addition, in the food industry, the use of cherry wood for smoking cheeses, such as Cheddar, is notable [8], as well as in smoking pork preparations [9]. However, this preservation method can promote contamination of food with carcinogenic polycyclic aromatic hydrocarbons [10].

Recent studies suggest that plant waste can significantly contribute to the development of sustainable alternatives in various industries, even as a substitute for lignocellulos. For example, Öncül et al. demonstrated the viability of replacing filler materials with woody biomass of *P. avium* to obtain environmentally sustainable polymeric materials. The results showed that the biocomposite containing only 5% filler material exhibits optimal mechanical properties [11].

Cherry trees and raspberries, plants belonging to the *Rosaceae* family, have a distinct distribution: genus *Prunus* ssp. is found across all continents except Antarctica, and genus *Rubus* ssp. is mainly distributed throughout the temperate zone of the northern hemisphere. These species, easily adaptable to different environmental conditions (climate, soil, etc.), with a variable chemical composition depending on numerous factors such as cultivation techniques, region, ripening, harvesting, and storage, are well represented in the northeastern area of Romania [12].

Plant waste from species such as red raspberry (*Rubus idaeus*) and cherry trees (*Prunus avium*—sweet cherry, *Prunus serotina*—bitter black cherry, *Prunus cerasus*—sour cherry) exhibits significant pharmacological potential, representing a path for sustainable and ecological valorization. Red raspberry stems contain compounds with antioxidant, anti-inflammatory [13], and antitumoral [14] actions, making them attractive to the pharmaceutical industry. According to specialized literature, calcium and magnesium are the most representative minerals, in terms of quantity, found in red raspberry stems [15]. Additionally, the twigs of bitter black cherry and sweet cherry are rich in phytochemical compounds [16] with beneficial effects on health, including antioxidant and anti-inflammatory activities [17,18]. These plant wastes can be transformed into valuable ingredients in the pharmaceutical and cosmetic industries, having applicability in medical treatments as well as in the development of new products [19].

Up to now, all the results suggest that *Rubus* and *Prunus* wastes possess significant potential for further research to generate value-added products which can be applied in multiple industries, offering both economic and health benefits.

However, there is a need for more in-depth research to fully understand the potential of these resources and to optimize the extraction and utilization process of bioactive compounds.

This review summarizes the research progress related to the bioactive compound content of certain *Rubus* and *Prunus* species, their extraction and isolation, as well as information regarding their pharmacological applicability and limitations. Also, aspects presented in the specialized literature regarding the structure–activity relationship of bioactive compounds and the synergistic effect they can have are highlighted, which could open the way to new applications.

## 2. Research Methodology

The selection of specialized literature was carried out with particular care, applying clear criteria to guarantee both thematic relevance and the scientific quality of the sources used in the following databases: PubMed, Science Direct, and SpringerLink.

Only those studies that specifically explored the chemical composition, the possibilities of valorization, and the practical uses of plant waste from the four investigated species were included in the analysis, with a special emphasis on twigs and shoots—plant residues frequently resulting from maintenance or harvesting activities. In addition, priority was given to works that provided detailed characterizations of the plant material and/or proposed solutions applicable in various fields, such as the pharmaceutical, food, cosmetic, or agricultural industries.

The initial documentation generated a considerable volume of information, exceeding 50,000 scientific articles. A large part of this resulted from the combination of different search terms such as:

- the expression “*Rubus idaeus* + shoots + bioactive compounds” produced over 1570 relevant results;
- the search “*Prunus avium* + twigs + bioactive compounds” provided approximately 2400 studies;
- “*Prunus serotina* + twigs + bioactive compounds” led to the identification of 540 articles;
- and the formula “*Prunus cerasus* + twigs + bioactive compounds” returned more than 1000 works.

More general terms, such as plant waste, twigs, biomass, or extraction of phenolic compounds, generated a very large number of results (over 26,000 for prunus woody biomass and over 16,000 for *Rubus* woody biomass). However, only those sources that were directly related to the species analyzed were retained for evaluation.

The narrowing of the search period was determined by the relatively recent interest of researchers in the sustainable valorization of plant waste. Also, the desire to present the latest experimental observations regarding the extraction and isolation of bioactive compounds, which are also viable from an ecological point of view, was also the basis for limiting the search interval.

**Figure 1:**
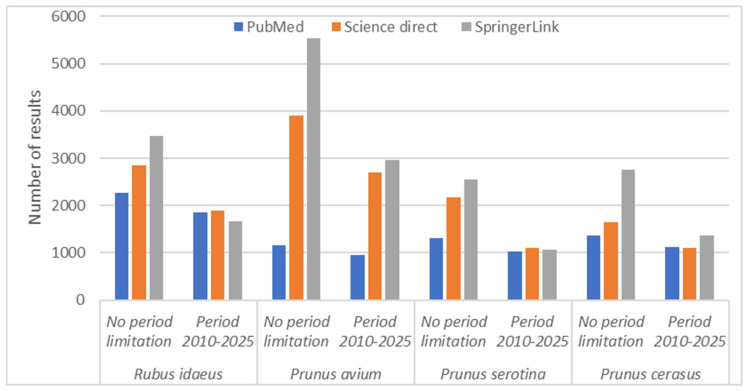
The distribution of articles by species published between 2010 and 2025.

Considering the graph presented in Figure 1, there is an upward trend in recent years in the interest shown by specialists in the field for the four previously mentioned species.

After applying all the selection filters-which targeted the relevance of the subject, methodological clarity, applicative value, and the elimination of redundancies-over 300 scientific sources were finally retained, with less than 10% of these published before 2010. This rigorous approach was the basis for a well-founded synthesis, which highlights the real potential for valorization of twigs and shoots from the *R. idaeus*, *P. avium*, *P. serotina*, and *P. cerasus* species, contributing to the promotion of sustainable practices and the consolidation of the circular economy in the agricultural and industrial sectors.

## 3. Bioactive Compounds from Plant Waste of *Rubus idaeus*, *Prunus serotina*, *Prunus avium*, and *Prunus cerasus*

From both an ecological and economic perspective, plant waste, a diverse mixture of woody and vegetable by-products generated mainly through deforestation and landscape maintenance, orchards, etc., requires appropriate management to transform it into a valuable and sustainable resource.

It is worth noting that woody biomass, a heterogeneous material, which is mainly considered a source of three natural polymers (cellulose, hemicelluloses and lignin), also contains an extractable fraction, which, although minor (<9%), includes important classes of compounds with potential biological activity (terpenoids, phenolic compounds, tannins, glycosides, vitamins, fatty acids, minerals, etc.).

**Figure 2:**
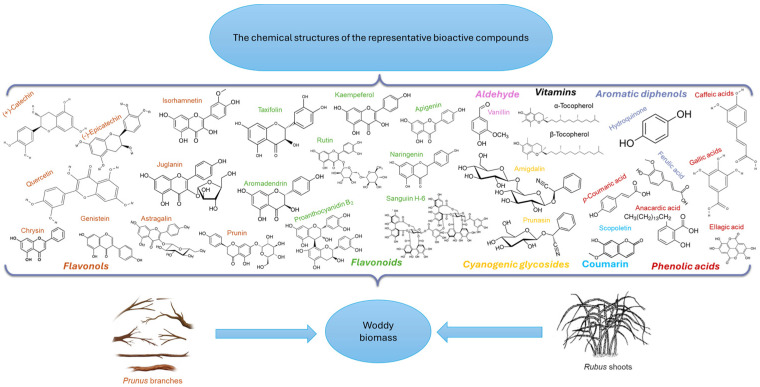
The chemical structures of the representative bioactive compounds from *Prunus* and *Rubus* woody biomass with health benefits.

A recent study by Newman and Cragg [20] on the impact of natural substances on pharmaceutical products introduced on the market between 1981 and 2019 highlights that over 37% of the 1881 molecules marketed during this period are based on natural sources: 71 are strictly natural, 356 are derivatives of natural substances obtained by semi-synthesis, and 272 are obtained by organic synthesis, having a pharmacophore inspired by natural substances. These data confirm the importance of focusing on the efficient valorization of the vegetative parts of *Rubus* and *Prunus* as sources of bioactive compounds (Figure 2).

The studied literature showed that there is a relative similarity in the chemical composition of the three *Prunus* species, but specific bioactive compounds have also been identified, such as juglanin [17] and prunasin [19] in *P. serotina*, taxifolin and vanillin [7] in *P. avium*, and phlorizin [21] and galangin [22] in *P. cerasus*. On the other hand, *R. idaeus* stands out due to its content of sanguiin H6 [23] and casuarinin [24]. Among the biologically active chemical compounds identified in all plant sources are caffeic acid and p-coumaric acid, among others [25,26,27].

In the following table, several bioactive compounds found in the four plant sources are presented, for which concentrations have also been identified in the specialized literature.

According to the data presented in Table 1, it is observed that *R. idaeus* exhibits a higher concentration of bioactive compounds common with *Prunus* species, but there are also exceptions, such as chlorogenic and *p*-coumaric acids, which are found in higher concentrations in *P. avium*, as well as ferulic acid, which has a higher concentration in *P. serotina*.

The polyphenol concentration was investigated in 11 varieties of *R. idaeus*; the presence of pentoside quercetin was identified in a single variety, with a concentration of 23.9 g/100 g dry shoot sample, while the compound sanguiin was identified in 10 out of 11 varieties [23]. The rarity of quercetin pentoside may be justified by different climatic growing conditions or the earlier character of the variety. A specialized study aimed at identifying the impact of covering fruit trees with anti-hail netting on the phytochemical profile revealed that direct exposure to sunlight can lead to a lower content of quercetin pentoside compared to the content when the crop is covered [33]. The stems are notable for their content of chlorogenic acid and proanthocyanidin B1, but smaller amounts of catechin, salicylic acid, and astragalin are also present [15]. The dominant component in the leaf extract of *R. idaeus* is casuarinin, which constitutes 83% of all identified polyphenolic compounds in the extract [24]. Although the studies consulted did not explicitly highlight the presence of casuarinine in the extracts, it was noted that ellagitannins, a class to which casuarinine also belongs, are found in high concentrations in the leaf extract [34].

In contrast to *Rubus*, *Prunus* species show divergent trends in phenolic distribution. The total phenolic content, expressed in mg gallic acid/g dry sample, is significantly higher in *P. avium* branches (301.98) compared to leaves (100.71) or flowers (81.2). On the other hand, the flavonoid content is lower in branch samples, whether it is hydroethanolic extract or infusion [28].

Among the organic acids mentioned by various studies [31,35], the significant presence, both in fruits and in plant waste, of malic and quinic acids is noteworthy, while tartaric, citric, succinic, or oxalic acids showed a marginal content.

In a study that includes a comparative chemical analysis of the fruits and branches of *P. avium*, it was highlighted that the amount of oxalic acid in the branches is double that of the content in cherries, and the citric acid content is approximately 35 times higher in the branches. Significant differences were also identified in the tocopherol content, with *γ*-tocopherol being identified only in the branches [31].

Wojdyło et al. [35] demonstrated through the analysis of extracts obtained from fruits and leaves of *P. avium* and *P. cerasus* that there is a higher concentration of polyphenolic compounds in leaves compared to fruits, regardless of the analyzed variety. However, it is worth mentioning that anthocyanins were identified only in fruit extracts.

The polyphenolic compounds in the leaves were quite diverse; therefore, it can be considered that the leaves may represent an alternative, unconventional, and cheap source of antioxidants and preservatives, with various uses in the pharmaceutical, cosmetic, and food industries [35].

Overall, these results (Table 1) clearly indicate that the accumulation of bioactive compounds is closely dependent on the plant part analyzed.

Also, the differences between the results reported in various studies regarding the presence/absence of some compounds and their quantity could be explained by the stage of development of the analyzed parts, as well as by the different applied extraction/separation and identification methodologies.

## 4. Extraction and Isolation Methods

### 4.1. Extraction

The methods for extracting bioactive compounds from the woody biomass of various trees and shrubs are extremely diverse. The literature highlights that a number of solvents used for the extraction of compounds from different parts of berry plants are also effective for red raspberries: acidified ethanol–water extraction to obtain anthocyanidins, procyanidins, or caffeoylquinic acids [36]; methanolic extraction to obtain polyphenolic compounds and flavonoids; acetone–water mixture extraction to isolate tannins and methanol-hydrochloric acid solution for anthocyanins [37]. Considering that some extracts may have applicability in the food industry, the use of solvents such as methanol is not safe; therefore, the adoption of green technologies, such as subcritical water extraction, is necessary [38].

Regarding the extraction of bioactive compounds from cherry branches, a study comparing conventional extraction with ethanol (70 °C) to accelerated extraction (150 °C), also using ethanol as a solvent, concluded that the extraction temperature affects the quantity of bioactive compounds obtained more than the percentage of ethanol used. [39].

On the other hand, in light of the use of green extraction technologies for cherry plant parts, data from the literature has noted the use of supercritical carbon dioxide extraction [40], a technique that can also be applied to cherry woody biomass [41] or other plant samples [42].

To optimize this method, one can also resort to coupling it with microwave-assisted extraction. The application of the two combined techniques led to the optimization of extraction under the following conditions: particle size of approximately 0.3–0.4 mm, liquid–solid ratio of 54 mL/g, and extraction time of 30 min [43].

Table 2 presents the representative extraction methods applied to the four plant sources studied.

According to the data presented in the previous table, it is observed that, at least in the case of phenolic compounds, for all four plant sources, the extraction using ethanol or methanol as a solvent is among the most commonly used, also having satisfactory yields. Even if the extraction process can be optimized by using modern green methods, the presence of the alcoholic solvent could ensure performance regardless of the nature of the plant source.

The content of ellagic acid obtained in the extract from aerial parts of *R. idaeus* varies depending on the method used, as follows: 3.24 mg/g extract for decoction (solvent: water), 3.12 mg/g extract for infusion (solvent: water), 2.45 mg/g extract for maceration (hydroalcoholic solvent), and 2.38 mg/g extract for ultrasound-assisted extraction (hydroalcoholic solvent) [47]. The same study mentions that for phenol extraction, ultrasound-assisted extraction is more efficient, while for flavonoids, infusion and maceration are recommended. Knowing that the solubility of ellagic acid in water and alcohol is influenced by temperature, probably, these results obtained, better in the case of extraction by decoction and infusion, are due to the different working conditions: the high temperature of the decoction and infusion versus UAE in an ice bath and maceration at ambient temperature, respectively. Among the natural sources of ellagic acid, some studies mention residues from the wood industry as an attractive alternative in terms of commercial exploitation. But at the same time, it is emphasized that it is necessary to find an ecological extraction methodology, knowing that currently the production of commercial ellagic acid from natural sources is based on obtaining extracts from plants rich in ellagitannins using acid–methanol mixtures as solvents, followed by their hydrolysis in a strongly acidic environment (HCl or concentrated H_2_SO_4_) [54].

A study conducted in 2021 by Brozdowski et al. demonstrated that in the case of phenolic compounds extracted from dried cherry leaves/flowers (*P. serotina*), the content was significantly higher when methanol was used as the solvent, compared to aqueous extraction [27]. Ademović et al. showed that the use of ethanol as a solvent led to an increase in the total content of phenols and flavonoids compared to the aqueous extraction of dried *P. avium* branches, achieving concentrations 3 and 4 times higher in the first case, respectively [18]. Taking into account the field in which these extracts are intended to be utilized, even if the extraction yield when using methanol or alcohol has proven to be more efficient than an aqueous medium, it is desirable to use eco-friendly solvents.

Regarding the chemical composition of the branches of *P. avium* and *P. cerasus*, there are no significant differences in the case of extracts obtained with subcritical water, which also supports the relatively similar biological activity [55].

### 4.2. Isolation

The main techniques for refining and fractionating crude extracts in order to isolate one or more groups of bioactive molecules found in *Rubus* and *Prunus* species are presented in Table 3.

The data presented in the previous table highlight chromatographic methods as the most frequently used in the isolation of the bioactive compounds targeted in this study. Additionally, their lack of or low affinity for water is highlighted, and thermolabile or thermostable compounds appear in the isolate (Table 3). The literature has highlighted the fact that aglycone flavonoids degrade faster under the influence of temperature, compared to glycosylated ones [92]. It is important to mention the concern for a reproducible isolation and the application of specific structural confirmation methods (^1^H NMR, ^13^C NMR, HMBC, HSQC, COSY, NOESY, MS, UV absorption spectroscopy, etc.), robust and accurate to preserve the quality and activity of the phytocompounds and the extracts obtained.

## 5. Biological Activity

### 5.1. Pharmacological Importance, Bioavailability, and Toxicity

The biological activity of red raspberry, sweet cherry, sour cherry, and black cherry extracts can be determined both by the presence of common bioactive compounds identified in the four plant sources described previously, as well as by those specific to each species. Table 4 summarizes the information from the literature regarding their pharmacological activity, bioavailability, and toxicity.

**Figure 3:**
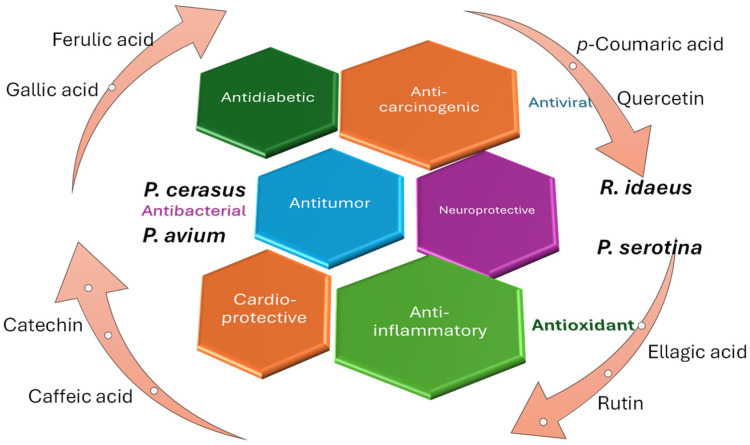
Pharmacological and pro-health importance of several common compounds identified in *Rubus* and *Prunus* species.

As highlighted in the previous table, the bioactive compounds from the species *R. idaeus*, *P. serotina*, *P. avium*, and *P. cerasus* exhibit multiple bioactive activities in the medical or dermato-cosmetic field. Their applicability in skincare products is highlighted by the antioxidant capacity exhibited by many compounds, but they also display more complex activities, such as anticancer or neuroprotective effects (Figure 3).

For a product containing bioactive compounds to be declared effective, it is necessary to know both its bioavailability and its toxicity [251].

Despite the biological activities possessed by the compounds identified in the *Rubus* and *Prunus* species, some exhibit toxicity to the human body, as can be seen in Table 4. This aspect of toxicity necessitates a series of additional studies to fully understand the interactions between bioactive compounds, with the aim of identifying methods to mitigate or eliminate these adverse reactions, for the maximum enhancement of pharma-co-logical effects.

### 5.2. Synergistic Activity

Compounds such as salicylic acid and astragalin from *R. idaeus* [15]; juglanin and isorhamnetin from *P. serotina* [17,139]; taxifolin, chrysin, and vanillin from *P. avium* [7,22]; phloridzin and rutin from *P. cerasus* [21,29] could exhibit synergistic action, which would enhance the individual pharmacological activities.

Interest in synergistic actions between bioactive substances has increased in recent years due to recent paradigm shifts. In this context, Table 5 presents several synergistic actions of the aforementioned bioactive compounds, based on bibliographic references consulted for this study.

These findings suggest the need to continue investigations on the possibility of using extracts obtained from the four sources, in different mixtures, in order to improve their biological activity (anticarcinogenic, anti-inflammatory, antioxidant, etc.), to enhance the effect of certain medications or medical procedures, and/or minimize the adverse effects associated with the treatments.

### 5.3. Structure–Activity Relationship Study

Considering the multiple biological activities of the compounds present in the four investigated sources, data from the literature regarding the structure-activity relationship were taken into account, which can help to understand how the chemical structures of these molecules influence their biological activities.

#### 5.3.1. Phenolic Compounds

Phenolic compounds exhibit antioxidant activity, among other things, which is determined by their ability to donate hydrogen atoms or electrons, but it can also be attributed to the possibility of metal chelation [269]. Additionally, studies in the field have shown that the antioxidant activity of phenols is influenced by the presence and position of the hydroxyl group [269,270].

Hydroxybenzoic acids exhibit a closer relationship between their antibacterial activity and hydrophobicity compared to hydroxycinnamic acids [271]. It is worth mentioning that this activity is influenced by the presence of hydroxyl groups, as well as by that of double bonds [272].

Antioxidant capacity assessment (ORAC assay) showed that extracts rich in polyphenolic and flavanol compounds, such as those from *P. cerasus* leaves and fruits, compared to those from *P. avium*, are useful for both cosmetic and pharmaceutical use [35].

1. Simple Phenols

- Hydroquinone, in the phenolic structural form, called 1,4-dihydroxybenzene, due to the presence of the hydroxyl group in the para position, exhibits antioxidant activity [273].

2. Flavonoids

Some flavonoids have the ability to inhibit monoamine oxidase, an enzyme responsible for the onset of certain neurological disorders. An experimental study demonstrated that this inhibitory activity exhibits an ascending character for the following series of flavonoids: apigenin, luteolin, quercetin, aromadendrin, and taxifolin [274].

The presence of hydroxyl groups in the structure of flavonoids influences their antidepressant activity, specifically, the groups in positions 2 and 4 support this activity [275]. Additionally, the number of groups is particularly important; proanthocyanidins, which are polymerized monomers and therefore contain more hydroxyl groups, exhibit higher antioxidant activity, whereas the glycosylation of flavonoids reduces this activity [276]. On the other hand, methoxy or glycosidic groups attached to the flavonoid structure reduce their antioxidant activity [277].

- Quercetin, named 2-(3,4-dihydroxyphenyl)-3,5,7-trihydroxy-4H-chromen-4-one [278], whose basic structure is represented by two phenyl groups connected by three carbon atoms, can be arranged in an open form or in the form of a heterocyclic ring [279]. The antioxidant activity of this compound is determined by the presence of the hydroxyl group, which is why some quercetin derivatives exhibit lower activity. On the other hand, obtaining methylated derivatives can lead to an increase in anti-inflammatory activity, and through glycosylation reactions, compounds with higher bioavailability regarding the antiobesity effect can be obtained [280]. Of the five hydroxyl groups in the structure of quercetin, only those at positions 3, 3′, and 4′ are responsible for the antioxidant activity of this compound, also being involved in its photolability [84]. The presence of the double bond in the heterocyclic ring of quercetin determines the manner in which this compound binds to DNA, by fitting into the helix of deoxyribonucleic acid, compared to naringenin, which does not have that double bond and exhibits a groove-type DNA binding [281]. The inhibitory activity on lipase is influenced by the structure of flavonoids as follows: it decreases through the hydrogenation of the double bond in the C ring, specifically through the glycosylation reaction, and increases with the presence of the carbonyl group or the hydroxylation reaction. Quercetin, due to its chemical structure, exhibits this activity, but it is lower than that of luteolin [282].
- Astragalin is also known as kaempferol 3-*O*-*β*-d-glucopyranoside. The substitution of phenolic hydroxyl groups influences the anti-inflammatory activity of astragalin, having a stronger effect than chrysin or luteolin [115].
- Rutin, known as 3′,4′,5,7-tetrahydroxyflavone-3-rutinoside or quercetin-3-rutinoside, is a flavonoid glycoside formed from quercetin and rutin [87]. The antioxidant activity of rutin can be enhanced by complexation with cyclodextrin [283]. On the other hand, glycosylation of this compound leads to an increase in antioxidant, antibacterial, and *α*-glucosidase inhibitory activities [88].
- Aromadendrin contains four hydroxyl groups in its structure and is also called (2*R*,3*R*)-3,5,7-trihydroxy-2-(4-hydroxyphenyl)-2,3-dihydrochromen-4-one. This compound exhibits multiple pharmacological activities, but in the case of antidiabetic and anticancer actions, the 7-O methylated derivative stands out, while methylation at the 4′-O position is noted to be effective for antiulcer activity [157].
- Juglanin (kaempferol 3-*O*-*α*-L-arabinofuranoside) contains multiple hydroxyl groups in its structure. This compound exhibits a lower antiradical effect compared to quercetin, the scientific justification being the presence of a single hydroxyl group on ring B [17].
- Kaempferol, also named 3,5,7-trihydroxy-2-(4-hydroxyphenyl)-4H-1-benzopyran-4-one, has a diphenylpropane structure and can be obtained through a series of reactions applied to naringenin [134,135]. In the study conducted by Rho et al., it was highlighted that depigmentation activity and cytotoxicity are enhanced by the presence of the hydroxyl group at position 3 [284].
- Isorhamnetin is a flavonoid, considered a methylated derivative of quercetin [261]. The methoxy group at position 3′ is associated with the antitumor activity of this compound [70].
- Prunin, a flavanone glycoside, is obtained following the hydrolysis process of naringenin. In the case of prunin laurate, a strong antibacterial activity against *Porphyromonas gingivalis* was shown by Wada et al. [285]. Additionally, in another study, when examining naringenin derivatives, it was highlighted that an aliphatic chain of 10–12 carbon atoms attached to ring A has the ability to enhance antimicrobial activity, with alkylprunin being an important representative [286].
- Apigenin or 4′,5,7–trihydroxyflavone, contains a 2-phenylchromen-4-one skeleton [63]. A study aimed at comparing the biological activity of apigenin and one of its derivatives, apigenin-7-*O*-glucoside, concluded that the presence of the sugar moiety in the derivative resulted in stronger antifungal activity against *Candida albicans* and *Candida glabrata*. Additionally, in vitro, the glycosidic derivative exhibits higher cytotoxic activity against cancer cells in the case of colon cancer, compared to apigenin [287].
- Chrysin (5,7-dihydroxyflavone) is a flavone that contains hydroxyl and keto functional groups [176]. The antioxidant activity of this compound is correlated with the lack of hydroxyl in rings B and C, as well as the presence of the carbonyl group on C4 and the double bond between C2 and C3 [288]. Liu et al. highlighted that halogenated derivatives exhibit stronger anticancer activity. Additionally, an enhancement of the effect was observed when the C7-OH of ring A was linked to various hydrophilic amines. Regarding the anti-inflammatory activity, a strong effect was demonstrated in the case of the derivative containing a cyclic pyridine at position 8 [289].
- Naringenin has two hydroxyl groups missing in its chemical structure compared to quercetin, which explains its lower antioxidant activity. Quercetin, on the other hand, has an antioxidant effect comparable to that of vitamin C, and the presence of two hydroxyl groups on ring C, instead of one as in the case of naringenin, leads to the formation of a stabilized quinone structure that contributes to enhancing the effect [281]. The antibacterial activity of naringenin is lower than that of other flavones that contain fewer hydroxyl groups; additionally, the position of these groups also influences the activity, so compounds that have hydroxyl groups in ring A but not in ring B exhibit significant activity. Methylation of hydroxyl groups may contribute to the reduction of the antibacterial effect [290].
- Taxifolin exhibits inhibitory activity against certain protein structures, such as amyloid fibrils, which have been highlighted in the literature as being responsible for the onset of Alzheimer’s disease. This inhibitory activity is due to the presence of the catechol group in ring B [291].
- Catechin is a flavan-3-ol, also named (2*R*,3*S*)-2-(3,4-dihydroxyphenyl)-3,4-dihydro-2H-chromene-3,5,7-triol. The position and number of hydroxyl groups influence the antibacterial activity of catechins. Additionally, the polymerization of catechin molecules enhances activity, as is the case with theaflavins [292]. The antioxidant activity is correlated with the presence of the hydroxyl group in position 3 [293].
- Genistein (4′,5,7-trihydroxyisoflavone) is a phytoestrogen that can be synthesized from naringenin in plants. It exhibits characteristics similar to those of the estrogen estradiol-17β, due to structural similarities consisting of the presence of the phenolic ring and the distance between the hydroxyl groups [294].
- Phlorizin, phloretin 2′-*β*-D-glucoside, according to Li et al., exhibits lower antioxidant activity than the parent compound because the glycosylation reaction reduces the number of phenolic hydroxyl groups [295].

Guerrero et al. investigated several flavonoids in terms of their inhibitory activity on the angiotensin-converting enzyme and highlighted the fact that at the structural level, there are some key elements underlying this activity, such as the presence of the catechol group at the B ring, the ketone group found at C4 in the C ring, and the double bond be-tween C2 and C3 of the C ring. It was concluded that this inhibitory activity has an upward trend for the following compounds:

- at a concentration of 500 µM: hesperetin, genistein, epicatechin, naringenin, apigenin, kaempferol, quercetin, and rutin;
- at a concentration of 100 µM: quercetin, rutin, kaempferol, and luteolin.

It should be mentioned that the highest activity was recorded at the minimum concentration [296].

Zhang et al. investigated the structure–activity relationship of some flavonoids responsible for inhibiting a breast cancer-resistant protein and concluded that, in this case as well, the double bond between C2 and C3 of ring C influences this interaction. Additionally, the importance of the B ring position, hydroxylation at position 5, and the absence of the hydroxyl group at position 3, as well as hydrophobic substitution at positions 6, 7, 8, or 4′, were mentioned as elements responsible for this protein–flavonoid interaction [297].

3. Tannins

- Proanthocyanidins contain significant monomeric units of flavan-3-ol, such as epicatechin or catechin, and are also referred to as condensed tannins. According to studies, these monomeric units influence biological activity, such as antioxidant or antidiabetic activity [247,298].
- Sanguiin H6, an ellagitannin derived from ellagic acid, has multiple biological activities that are influenced by the presence of hydroxyl groups and the galloyl configuration [299].

4. Phenolic Acids

A carboxyl group derived from an acid and at least one hydroxyl group that replaces hydrogen atoms in the benzene rings gives rise to phenolic acids [298].

The antioxidant activity of hydroxycinnamic acids is directly positively influenced by the presence of a double bond in the side chain, the number and position of hydroxyl groups on the aromatic ring, the esterification or amidation of the carboxyl group, as well as the presence of the catechol group [299].

- 3,4-Dihydroxycinnamic acid exhibits hepatoprotective activity that can be enhanced through methoxylation at positions 3 or 4 [300]. The esterification gives rise to derivatives that exhibit remarkable antileishmanial activity. Otero et al. highlighted in a study that the bioactivity of cinnamic acid derivatives depends on the degree of oxygenation at positions 3 and 4, the presence of a double bond in the side chain, and hydroxyl groups, as well as the length of the alkyl chain [301].
- Caffeic acid is a hydroxycinnamic acid that contains an aromatic ring and three hydroxyl groups, along with the double bond in the carbon chain, with anticancer activity [212]. Anilides and aliphatic amides of caffeic acid enhance its antioxidant activity [302]. The attachment of a naphthyl ring increases the capacity of caffeic acid to inhibit monoamine oxidase, an enzyme responsible for multiple neurological disorders [301,303].
- *p*-Coumaric acid, a phenolic acid, derived from cinnamic acid, is also known as 4-hydroxycinnamic acid [304]. Derivatives of *p*-coumaric acid exhibit higher antimicrobial activity, especially esters, anilides, and amides with bulky aromatic groups [305].
- Ferulic acid, also called 4-hydroxy-3-methoxycinnamic acid, is responsible for some biological activities [306]. The anticancer activity of some ferulic acid derivatives was investigated; thus, although some derivatives exhibit lower activity compared to caffeic acid derivatives, the phenylsulfonylfuroxan nitrates of ferulic acid stand out as having strong anticancer activity [307]. Ferulic acid, found in raspberry plant parts, has the ability to stabilize anthocyanins, but it is also recognized for its involvement in flavonoid catabolism, particularly in the spontaneous carboxylation of caffeic acid [308].
- Chlorogenic acid is derived from caffeic acid and quinic acid, and the hydroxyl groups present in its structure are responsible for the strong antioxidant effect it exhibits [58].
- Ellagic acid or 2,3,7,8-tetrahydroxy [1]-benzopyrano [5,4,3-cde] benzopyran-5,10-dione, structurally contains a hydrophilic part, represented by phenolic groups and lactone-type groups, as well as a lipophilic part represented by the four phenolic rings [50…60]. The anticancer activity of this compound is closely related to its chemical structure, specifically the presence of hydroxyl groups at positions 3 and 4, as well as the presence of lactone groups [309,310].
- Salicylic acid or 2-hydroxybenzoic acid is a plant hormone, being the main precursor of aspirin. From a structural perspective, it is notable for the *ortho* arrangement of the hydroxyl and carboxyl groups [311]. The inhibition of luciferase by salicylic acid is enhanced by the amidation of the carboxyl group or the substitution of chlorine at position 5 [312].
- Anacardic acid, a derivative of salicylic acid, has a side chain with different degrees of unsaturation, which is responsible for its varied biological activity. Regarding antioxidant activity, trienic anacardic acid (15:3) stands out, while for antifungal activity, monoenic anacardic acid (15:1) is highlighted [313]. The biological activity of anacardic acid is closely related to the structure of the side chain; thus, the presence of the trienic alkyl side chain determines a strong bactericidal activity against *Streptococcus mutans* and *Staphylococcus aureus*, while the saturated alkyl chain acts against *Propionibacterium acnes*. The antioxidant activity is synergistically influenced by the length of the alkyl chain, the presence of the salicylic acid moiety, as well as the stereochemistry of the side chain [56]. Some researchers have noted that the anticancer activity of anacardic acid largely depends on the molecular volume of the hydrophobic side chain, in addition to its metal-chelating ability and its action as a surfactant [93].

#### 5.3.2. Coumarins

- Scopoletin, 6-methoxy-7-hydroxycoumarin, is characterized by the presence of a single hydroxyl group, a methoxy group, and a keto group [210]. Liu et al. demonstrated that derivatives containing a Δ3,4 olefinic bond, as well as naphthyl or phenyl groups with a sulfate ester at the C7 position, enhance insecticidal activity against *Tetranychus cinnabarinus* and *Artemia salina*, respectively [314].

#### 5.3.3. Cyanogenic Glycosides

- Prunasin, the glucoside of (R)-mandelonitrile, can be glycosylated with the formation of amygdalin, and it can be converted into mandelonitrile by *α*-glucosidase or a hydrolase, and subsequently hydrolyzed into benzaldehyde and hydrocyanic acid [315].

#### 5.3.4. Aldehyde

- Vanillin is an important flavor molecule, being named 4-hydroxy-3-methoxybenzaldehyde, and constitutes the major component of vanilla [316]. The aldehyde group in the structure of vanillin, as well as the position of the side group on the benzene ring, supports the antifungal activity exhibited by this compound [317]. Furthermore, this compound also exhibits antioxidant activity, stronger than that of ascorbic acid, justified by its self-dimerization in contact with free radicals [318].

#### 5.3.5. Terpenoid

- Squalene is a precursor of cholesterol, and not only a triterpene that contains 30 carbon atoms in its structure. In the synthesis of cholesterol, the process was initially proposed to be described as a cyclization of squalene to lanosterol; later, it was demonstrated that it oxidizes to form monooxidosqualene before cyclization [319]. It exhibits a high detoxification capacity due to its ability to attach to uncharged substances, owing to its nonpolarity [320].

#### 5.3.6. Vitamins

- Ascorbic acid, better known as vitamin C, is a compound with multiple bioactive activities, including antioxidant activity. This activity is justified on one hand by the acid’s ability to donate single hydrogen atoms, and on the other hand by the interaction between radicals and the monodehydroascorbate anion [321]. It is also worth mentioning the importance of vitamin C in collagen synthesis, a compound extremely important for human health, as well as in the fixation of vitamin E or iron [322]. The structure of lactone, with two ionizable hydroxyl groups, makes this compound an excellent reducing agent. It oxidizes successively, forming ascorbate radical and then dehydroascorbic acid, a mechanism that underlies many biological activities [323].
- Tocopherol, belonging to the vitamin E family, has a chemical structure that contains a polar chromanol ring and a lipophilic phytyl chain, and its antioxidant activity is justified by its ability to form tocopherol quinone [324]. Just like in the case of vitamin C, the presence of hydroxyl groups in the chemical structure of tocopherols, which act as hydrogen donors for peroxyl radicals, reveals other biological activities, such as cellular signaling properties [325].

Research in the field has highlighted the fact that *Rubus* and *Prunus* plant waste can be used to obtain extracts with broad applicability in the medical field. The antioxidant, antimicrobial, and anti-inflammatory actions have been explored both in vitro and in vivo. It is worth noting that the identified studies, both in vitro and in vivo, mainly reported on the leaf extract [326,327].

The reviewed literature underscores the importance of woody biomass of *Prunus* and *Rubus* species as a source of bioactive compounds with multiple pharmacological and cosmetic applications [328].

## 6. Conclusions

Plant waste, including red raspberry stems and cherry twigs, presents significant potential for the development of a sustainable valorization model through the lens of bio-active compound content. They are predominantly applicable in the pharmaceutical and cosmetic industries, thus contributing both to the reduction of industrial waste and to the enhancement of the sustainability of economic processes.

In this analysis, recent data from the specialized literature on the biological activity of the main compounds identified in the species *R. idaeus*, *P. serotina*, *P. avium* and *P. cerasus* are presented, emphasizing the following aspects:

- presentation of the bioactive compounds representative of these species and highlighting their extraction and isolation methodology;
- correlation between biological activity and their chemical structure, with emphasis on the possible synergistic action of some compounds common to the four species.

The diversity of available extraction technologies is noteworthy, from conventional methods such as ethanol extraction, to more environmentally friendly solutions such as subcritical water extraction and supercritical CO_2_ extraction that allow the obtaining of bioactive compounds with high yields.

In addition, an affinity for chromatographic methods for the isolation and purification of target compounds was observed, a fact justified both by the complexity of the chemical composition of the plant matrix and by the need to obtain high-purity products in accordance with the requirements of the pharmaceutical and cosmetic industries.

These aspects represent a starting point for in-depth research into the four plant sources rich in active phytochemical compounds, which can become a valuable source for the manufacture of pharmaceutical, cosmetic, and even food products.

## 7. Future Perspectives

In order to maximize the potential for valorization of plant waste, it is essential to continue exploring the correlation between the structure and biological activity, the mechanism of action, the bioavailability, and the potential toxicity of the identified compounds. It is also important to develop and improve technologies that increase the separation efficiency of bioactive compounds, applicable on an industrial scale and with low environmental impact.

In addition to these challenges, further developments are needed to increase the adoption of waste recovery technologies, economic feasibility, market potential, and policy incentives to support the superior recovery of plant waste.

## Figures and Tables

**Table 1:** Representative classes/bioactive compounds found in different plant parts of *R. idaeus*, *P. serotina*, *P. avium*, and *P. cerasus*.

Bioactive Compound	Source	Content	References
***Flavonols***			
Catechin	*P. serotina* (dry leaves)	605–2342 mg/kg	[27]
	*R. idaeus* (Willlamette: non-lignified dry shoots)	129.3 mg/100 g	[23]
	*P. avium* (wood)	0.32–30.15 mg/g	[7]
	*P. avium* (dry leaves and branches)	6.879 mg/g and 0.42–3.74 mg/g	[28]
	*P. cerasus* (resin)	1.91 mg/L	[22]
	*P. avium* (resin)	0.33 mg/L	[22]
Epicatechin	*R. idaeus* (non-lignified dry shoots)	10.9–85.3 mg/100 g	[23]
	*P. avium* (wood)	0.36 mg/g	[7]
	*P. avium* (dry branches)	0.0873–0.1102 mg/g	[28]
Quercetin	*P. avium* (resin)	1.77 mg/L	[22]
	*P. cerasus* (resin)	0.63 mg/L	[22]
Quercetin derivates	*R. idaeus* (non-lignified dry shoots)	10.3–67.4 mg/100 g	[23]
Quercetin 3-*O*-rutinoside	*P. avium* (dry branches)	404.39–767 µg/g	[29]
Quercetin 3-*O*-hexosides	*P. avium* (dry branches)	665.76–1025.78 µg/g	[29]
Chrysin	*P. avium* (resin)	1.57 mg/L	[22]
	*P. cerasus* (resin)	0.16 mg/L	[22]
Genistein (genistein-7-*O*-glucoside)	*P. avium* (dry branches)	0.42–3.74 mg/g	[28]
***Flavonoids***			
Kaempferol-3-*O*-rutinoside	*R. idaeus* (dry leaves)	37.92 mg/g	[24]
	*P. avium* (dry leaves and branches)	6.6 mg/g and 0.88 mg/g	[28]
Kaempeferol	*R. idaeus* (dry leaves)	1.88–82.28 g/100 g	[30]
	*P. cerasus* (resin)	1.04 mg/L	[22]
	*P. avium* (resin)	0.22 mg/L	[22]
Rutin	*P. cerasus* (resin)	0.22 mg/L	[22]
	*P. avium* (resin)	0.19 mg/L	[22]
Apigenin hidroxihexoside	*P. serotina* (dry leaves)	57.4–63.4 mg/kg	[27]
Apigenin	*P. avium* (dry branches)	0.033 mg/g	[28]
Aromadendrin	*P. avium* (wood)	0.08–4.54 g/kg	[7]
Aromadendrin-7-*O*-hexoside	*P. avium* (dry branches)	0.86–2.66 mg/g	[28]
Naringenin	*P. avium* (resin)	5.01 mg/L	[22]
	*P. cerasus* (resin)	4.73 mg/L	[22]
	*P. avium* (dry leaves)	0.74 mg/g	[28]
	*P. avium* (wood)	0.17–0.41 mg/g	[7]
	*P. serotina* (lyophilized leaves)	0.13 mg/100 g	[16]
Naringenin-7-*O*-hexoside	*P. avium* (dry branches)	1482.67–1940.77 µg/g	[29]
Taxifolin	*P. avium* (wood)	0.09–8.46 mg/g	[7]
	*P. avium* (dry branches)	0.19–0.79 mg/g	[28]
***Tannins***			
Proanthocyanidin B_1_	*R. idaeus* (Willlamette: non-lignified dry shoots)	229 mg/100 g	[23]
	*P. avium* (wood)	0.15 mg/g	[7]
Proanthocyanidin B_2_	*R. idaeus* (Willlamette: non-lignified dry shoots)	646 mg/100 g	[23]
	*P. avium* (wood)	0.72 mg/g	[7]
Proanthocyanidin dimer B type 2	*P. avium* (dry branches)	7149.5–8810.67 µg/g	[29]
Proanthocyanidin dimer type B	*P. avium* (wood)	3.65 mg/g	[7]
Proanthocyanidin trimer type B	*P. avium* (wood)	1.25 mg/g	[7]
Sanguiin H-6	*R. idaeus* (non-lignified dry shoots)	139.2–633.1 mg/100 g	[23]
***Cyanogenic glycosides***			
Prunasin	*P. serotina* (leaves)	59.49 mg/g	[16]
Amigdalin	*P. serotina* (leaves)	20.95 mg/g	[16]
***Vitamins***			
*α*-Tocopherol	*P. avium* (lyophilized branches)	512.58 µg/100 g	[31]
*β*-Tocopherol	*P. avium* (lyophilized branches)	31.94 µg/100 g	[31]
*γ*-Tocopherol	*P. avium* (lyophilized branches)	23.58 µg/100 g	[31]
***Aldehyde***			
Vanillin	*P. avium* (wood)	4.68 mg/g	[7]
	*P. avium* (dry branches)	0.079 mg/g	[28]
***Phenolic acids***			
Ellagic acid	*R. idaeus* (non-lignified dry shoots)	26.1–106.8 mg/100 g	[23]
	*P. avium* (wood)	0.27 mg/g	[7]
Chlorogenic acid	*R. idaeus* (Willlamette: non-lignified dry shoots)	177.4 mg/100 g	[23]
	*P. serotina* (lyophilized leaves)	29.5 mg/100 g	[16]
	*P. avium* (dry leaves)	17.06 mg/g	[28]
	*P. avium* (resin)	0.62 mg/L	[22]
	*P. cerasus* (resin)	0.27 mg/L	[22]
Gallic acid	*R. idaeus* (Willlamette: non-lignified dry shoots)	72.2 mg/100 g	[23]
	*P. serotina* (lyophilized leaves)	19.56 mg/100 g	[16]
	*P. avium* (dry branches)	0.041–0.05 mg/g	[28]
Caffeic acid	*R. idaeus* (dry leaves)	0.64–7.21 mg/g	[32]
	*P. serotina* (dry leaves)	75–158.8 mg/kg	[27]
Ferulic acid	*P. serotina* (lyophilized leaves)	185.3 mg/100 g	[16]
	*P. avium* (dry branches)	0.22–0.23 mg/g	[28]
	*R. idaeus* (dry leaves)	0.1–0.49 mg/g	[32]
*p*-Coumaric acid	*P. serotina* (lyophilized leaves)	103.6 mg/100 g	[16]
	*P. avium* (wood)	26.3 mg/g	[7]
	*R. idaeus* (dry leaves)	0.07–0.95 mg/g	[32]
	*P. avium* (dry branches)	0.038–0.161 mg/g	[28]

**Table 2:** Extraction methods of bioactive compounds, applied to different types of plant waste from *Rubus* and *Prunus* species.

Bioactive Compound/Class of Compounds	Sources	Extraction Methods	References
***R. idaeus***			
Non-extractable/bound phenolic compounds	leaves	Acid and enzymatic hydrolysis (especially for ellagic acid)	[44]
Phenolic compounds	dry, non-lignified shoots	Soxhlet, using chloroform and methanol	[23,45]
	dry leaves	Reflux extraction	[30]
	dry leaves	Aqueous extraction in a mass ratio of 3:1	[24]
	dry leaves	Extraction with acetone and trichloromethane	[32]
	lyophilized leaves	Alcoholic extraction (2 g sample in 80 mL 70% methanol) with stirring	[46]
	dried leaves and woody material	Ultrasound-assisted extraction, using 1 g of dried and ground sample, 30 mL of 80:20 ethanol–water solution	[47]
Polyphenols, catechins, hydroxycinnamic acids, flavonoids	shoots	Reflux extraction (solid–liquid)	[48]
Quercetin 3-glucosid and K_2_ vitamin	frozen leaves	Ultrasound-assisted extraction (solvent: methanol–acetonitrile–water solution in a volume ratio of 2:2:1)	[49]
Carotenoids	lyophilized leaves	Extraction with acetone and hexane (4:6 *v/v*)	[46]
***P. serotina***			
Phenolic compounds	dry leaves	Ultrasound-assisted methanol extraction	[27]
	lyophilized leaves	Aqueous extraction	[16]
Tannins	dry bark	Reflux extraction (25 g sample + 200 mL 90% ethyl alcohol + 200 mL glacial acetic acid)	[50]
***P. avium***			
Phenolic compounds	dry branches	Pressurized liquid extraction (ethanol–water) Supercritical fluid extraction (CO_2_)	[26]
	lyophilized branches	Hydromethanolic extraction (80/20 *v/v*)–decoction Infusion (distilled water)	[31]
	lyophilized leaves	Extraction with water/methanol/ascorbic acid/hydrochloric acid 37% (6.8:3:0.1:0.1; *v/v/g/v*), assisted by ultrasound	[35]
	dry bark	Ultrasound-assisted extraction (solvent: 80% aqueous ethanol solution)	[51]
Fatty acids, organic and phenolic acids, aromatic aldehydes, isoprenoids	dry branches	Subcritical water extraction	[52]
Hydroxycinnamic acids	salks and leaves	Maceration Supercritical fluid extraction–CO_2_	[53]
Caffeic acid	salks	Solvent extraction, maceration	[53]
Proanthocyanidin	salks	Accelerated solvent extraction	[53]
Catechin	salks	Solvent extraction, supercritical fluid extraction, ultrasound-assisted extraction	[53]
***P. cerasus***			
Phenolic compounds	lyophilized leaves	Extraction with water/methanol/ascorbic acid/hydrochloric acid 37% (6.8:3:0.1:0.1; *v/v/g/v*), assisted by ultrasound	[35]
Fatty acids, organic and phenolic acids, aromatic aldehydes, isoprenoids	dry branches	Subcritical water extraction	[52]

**Table 3:** Isolation methods applicable to some representative bioactive compounds.

Bioactive Compound	Isolation Methods	Observations	References
Anacardic acid	Obtaining the extract using supercritical carbon dioxide, followed by precipitation in the form of calcium anacardate, which, after treatment with hydrochloric acid, is converted back into anacardic acid	It is a thermolabile compound, and distillation under low pressure favors the acid thermal decomposition into cardanol	[56]
Chlorogenic acid	Surface imprint polymerization based on hyper-branched amino magnetic nanoparticles	Soluble in water It is a thermosensitive compound and easily oxidized	[57,58]
Ellagic acid	The use of cotton fibers grafted with graphene oxide promotes insulation through hydrophobic interaction, serving as a stationary absorbent	It is thermally stable Slightly soluble in water, alcohol, and ether Soluble in potassium hydroxide High solubility in pyridine	[54,59,60]
Apigenin	The hydroalcoholic, methanolic, or ethyl acetate fractions of the aqueous extract are subjected to column chromatography and preparative HPLC The methanolic extract is subjected to partitioning with ethyl acetate, followed by column chromatography on silica gel for separation, thin-layer chromatography for purification, and NMR spectroscopy for compound confirmation	Low solubility in lipophilic and highly hydrophilic solvents High solubility in phosphate buffers with pH 7.5 Low solubility in water	[61,62,63]
Astragalin	The ethyl acetate fraction is concentrated and isolated by column chromatography (TLC and HPLC) on silica gel, using a mixture of ethyl acetate, methanol, and water as the eluent	Solubility is reduced in water	[64,65]
Hydroquinone	The crude extract is loaded onto a silica gel column (ethyl acetate and hexane), followed by purification through semi-preparative HPLC (methanol-water)	Soluble in methanol, ether, and water It oxidizes in contact with air and light Significant thermal sensitivity	[66,67,68]
Isorhamnetin	High-speed countercurrent preparative chromatography	Low solubility in water Thermal stability	[69,70,71]
Juglanin	The alcoholic extract is subjected to Sephadex column chromatography on silica gel, using a mixture of chloroform, methanol, and water as the eluent	Great solubility in water	[72,73]
Kaempeferol	The alcohol is evaporated under vacuum from the methanolic extract, yielding an ethyl acetate fraction that is separated with n-hexane and subjected to vacuum liquid chromatography, followed by other chromatographic techniques (Sephadex column and TLC) until the target compound is isolated	Low solubility in water Thermal stability	[74,75,76]
Naringenin	Methanolic extraction followed by crystallization in water containing 14–15% dichloromethane	Low solubility in water Solubility in different solvents: ethyl acetate > isopropanol > methanol > n-butanol > petroleum ether > hexane	[77,78,79]
Proanthocyanidins	Sephadex column chromatography	Solubility varies directly proportionally with temperature; in alcohol, it decreases with increasing molecular weight; it exhibits shorter interaction times with tetrahydrofuran and ethyl acetate	[80,81]
Prunin	The methanolic extract is divided into several fractions, the one soluble in ethyl acetate is subjected to silica gel chromatography, a mixture of chloroform and methanol is used as the eluent, followed by a separation using Sephadex, with methanol as the solvent	Low solubility in lipophilic media	[82,83]
Quercetin	The chloroform fraction of the ethanolic extract is subjected to column chromatography on silica gel, using a mixture of methanol, chloroform, and ethyl acetate as solvents. The use of a mixture of formic acid, water, and methanol in a gradient system, through the HPLC-DAD-MS/MS method. The application of column chromatography on polyamide of the ethyl acetate fraction	Insoluble in water. Stability to light in concentrations greater than 10% Unstable when exposed to atmospheric oxygen Thermal stability	[61,76,84,85]
Rutin	Dichloromethane fractions or aqueous fractions with a higher rutin content are obtained, which are chromatographically separated on a Sephadex column with methanol as the mobile phase	Low liposolubility. Increases water solubility through glycosylation	[86,87,88]
Sanguiin H6	Quantification from the hydrolytic solution, by HPLC	Soluble in water Hydrolyzes in acidic or basic environments, giving rise to ellagic acid	[89]
Scopoletin	Chromatographic separation (TLC or HPLC) with elution in a mixture of methanol and other compounds (chloroform, acetonitrile, acetic acid)	It is soluble in water and stable in solution at a pH between 3 and 10, a stability that can be extended in time and pH range by the addition of methanol	[90,91]

**Table 4:** Correlation of bioactive compound–pharmacological activity, bioavailability, and toxicity [17,65,67,70,75,78,87,93,94,95,96,97,98,99,100,101,102,103,104,105,106,107,108,109,110,111,112,113,114,115,116,117,118,119,120,121,122,123,124,125,126,127,128,129,130,131,132,133,134,135,136,137,138,139,140,141,142,143,144,145,146,147,148,149,150,151,152,153,154,155,156,157,158,159,160,161,162,163,164,165,166,167,168,169,170,171,172,173,174,175,176,177,178,179,180,181,182,183,184,185,186,187,188,189,190,191,192,193,194,195,196,197,198,199,200,201,202,203,204,205,206,207,208,209,210,211,212,213,214,215,216,217,218,219,220,221,222,223,224,225,226,227,228,229,230,231,232,233,234,235,236,237,238,239,240,241,242,243,244,245,246,247,248,249,250].

Bioactive Compound		Pharmacological Activity	Ref	Bioavailability and Toxicity	Ref
***R. idaeus***					
***Phenols***	Anacardic acid	Bactericide, anticancerogenic, fungicide, insecticide, anti-termite, and molluscicidal Tyrosinase and urease inhibition	[93,94]	Physicochemical stability and low water solubility result in limited bioavailability One of the main culprits of cashew allergy is due to the presence of the carboxyl group and the unsaturated side chain May induce allergic contact dermatitis	[93,96]
		Prevention and treatment of breast cancer	[95]		
	Sanguiin H6	Antioxidant Anticancerigenic (breast)	[97]	Low upon oral administration, being stable in the acidic environment of the stomach, hydrolysis is possible in the intestinal environment It has no adverse effects	[100,101]
		Anti-inflammatory	[98]		
		Antiangiogenic	[99]		
***Flavonoids***	Quercetin ramnoside	Antioxidant and liver protection	[102,103,104]	Higher than quercetin Does not present any potential toxicity to animals	[107,108]
		Antivirals	[105]		
		Restoring the intestinal microbiota	[106]		
	Astragalin (kaempferol 3-glucoside)	Antidepressant	[109]	Low, structural modification by enzymatic synthesis is suggested Studies conducted to date have not revealed any toxic activity of this compound	[65,115]
		Hypoglycemic	[110]		
		Anti-inflammatory, antioxidant, neuroprotective, cardioprotective, antiobesity, antiosteoporotic, anticancer, antiulcer, and antidiabetic	[111,112]		
		Analgesic, procoagulant, antibacterial, antiallergic, and antihepatotoxic	[113]		
		Neuroprotective	[114]		
***Vegetal hormones***	Kinetin	Antioxidant	[116]	Good oral absorption. Is not mutagenic nor cardiotoxic	[118]
		Inhibition of colorectal cancer	[117]		
	Salicylic acid	Antimicrobial and anti-inflammatory	[119]	Higher bioavailability in intravenous form compared to oral administration Topical toxicity is rare	[121,122]
		Obtaining the 4-chloro-5-chlorosulfonyl salicylic acid derivative–diuretic agent	[120]		
***Vitamins***	Ascorbic Acid (Vitamin C)	Antioxidant, anticancer Wound healing	[123]	Intravenous bioavailability is higher compared to oral Does not show toxicity even in higher doses	[124,125]
	Tocopherol (Vitamin E)	Antioxidant, platelet anticoagulant	[126]	The absorption efficiency can be close to 80%, but it depends on many factors (pH, presence of proteins, etc.)	[127]
***P. serotina***					
***Phenols***	Hydroquinone (1,4 dihydroxybenzene)	Reduces hyperpigmentation (possible side effects) May cause ochronosis	[128,129,130]	Improved permeability may be associated with an increase in toxicity due to poor physicochemical stability The instability of this compound can lead to the formation of potentially carcinogenic products, but skin and eye side effects can also be recorded It has high toxicity for the aquatic environment and soil In human and animal organisms, it promotes the occurrence of cancer, damages DNA, and favors allergic immune responses	[67,131,132]
		Antiphotoaging	[131]		
***Flavonoids***	Kaempferol	Antimicrobial, anti-inflammatory, antioxidant, antitumor, cardioprotective, neuroprotective, and antidiabetic, anticarcinogenic	[133]	Low May react with iron and decrease bioavailability May decrease the action of anticancer drugs Possible genotoxic action	[75,135]
		Antifungal and antiprotozoal, hepatoprotective, renoprotective, gastroprotective, and antimutagenic	[134]		
		Effective in treating cervical cancer	[112]		
***Flavonols***	Juglanin (kaempferol 3-*O*-*α*-L-arabinofuranoside)	Anti-inflammatory, antioxidant, antifibrotic, antithrombotic, antiangiogenic, hepatoprotective, hypolipidemic, hypoglycemic	[17]	Good, especially in glycoside form. Low probability of presenting toxicity when ingested, but significant when applied topically.	[17]
		Renal protection	[136]		
		Antidepressant	[137]		
		Procoagulant effect	[138]		
	Isorhamnetin	Neuroprotective, cardioprotective, antioxidant, anti-inflammatory, and antiapoptosis	[139]	Higher than quercetin as a liver protector It has no adverse effects and reduces those associated with classic cancer treatment	[70,143]
		Antiobesity	[140]		
		Antiviral	[141]		
		Anticoagulants	[142]		
***Terpen***	Squalen	Antitumor, antioxidant, and emollient activity on the skin	[144]	High cutaneous availability	[146]
		Vaccine adjuvant	[145]		
***Cyanogenic glycoside***	Prunasin	Treating respiratory conditions (risk of toxicity–cyanide release)	[146]	Larger in the form of decoction. Cyanide can be eliminated through hydrolysis, the lethal dose of which is 0.5 – 3.5 mg/kg body weight	[151,152]
		Anticarcinogenic properties	[147]		
		Anti-inflammatory and antioxidant	[148]		
		Hepatoprotective and antifibrogenic	[149,150]		
***P. avium***					
***Flavonoids***	Taxifolin	Anticarcinogenic (minimal adverse effects) Anti-inflammatory, hepatoprotective, antioxidant, cardioprotective, antimicrobial, antiviral, antifungal, antiangiogenic, antihyperglycemic, antipsoriatic, anti-Alzheimer	[153,154]	Low bioavailability Compared to quercetin, this compound is not phototoxic.	[155,156]
	Aromadendrin	Anti-inflammatory, antioxidant, antidiabetic, antiproliferative, antimicrobial, hepatoprotective, and gastroprotective	[157]	Studies suggest that it is not a mutagenic compound, but may exhibit promutagenic activity	[159]
		Antityrosinase, neuroprotective, cardioprotective, antiviral, immunomodulatory, antiacetylcholinesterase, antiapoptotic, antityrosinase, neuroprotective, cardioprotective, antiviral, immunomodulatory, antiacetylcholinesterase, antiapoptotic	[158]		
	Naringenin	Antioxidant, antitumor, antiviral, antibacterial, anti-inflammatory, antiadipogenic, anticancer, antiproliferative, and cardioprotective anti-HCV (hepatitis C virus)	[160]	Small, but still larger than that of the plum There are no adverse reactions recorded when administered to humans. In amphibian embryos, it produced mutations or death in fairly low doses.	[78,164,165]
		Antidiabetic	[161]		
		Antifibrogenic	[162]		
		Neuroprotective, antidiabetic, antidepressant	[163]		
	Apigenin	Radioprotective and radiosensitive	[166]	Oral bioavailability approaches 30% and increases with coadministration with friedelin.	[169,170]
		Neuroprotective, antidiabetic, antidepressant, anti-insomnia	[167]		
		Hepatoprotective, renoprotective, cardioprotective, antimicrobial, dermatoprotective (anti-UV, antiaging, combats dermatitis and supports wound healing), antiarthritic Supports oral and ocular health	[168]		
	Prunin	Antioxidant, anti-inflammatory, anticancer, immune regulation, antiosteoporosis, antihypoxia, and protective effects for the lungs, liver and kidneys	[171]	Exhibits selective toxicity for cancer cells, but glycosylated derivatives develop lower toxicity on human cells. Glycosylation at position 7 is responsible for increasing bioavailability	[171]
		Antiviral effect (Human Enterovirus A17)	[172]		
		Antidiabetic	[173]		
		Antianxiety	[174]		
		Broad-spectrum antibacterial activity	[175]		
***Flavone***	Chrysin (5,7-dihydroxyflavone)	Anti-inflammatory, anticancer, antidiabetic, antirachitic, antiasthmatic, antidepressant, neuroprotective	[176]	Low in oral administration caused by poor absorption, metabolism and rapid elimination At a dose of 400–500 mg, it does not cause any notable adverse effects, but it is likely to induce liver toxicity at the cellular level and inhibit de novo DNA synthesis	[176]
		Antihypercholesterolemic, cardioprotective, antiepileptic, antiamyloidogenic, antiatherogenic	[177]		
		Antidiabetic, antioxidant, antihyperlipidemic	[178]		
	Genistein	Effects of reducing the risk of osteoporosis and post-menopausal symptoms, as well as anticancer, antioxidant, cardioprotective, antiapoptotic, neuroprotective, hepatoprotective, and antimicrobial activities.	[179]	It grows in glycosylated form. Minimal toxicity at doses up to 16 mg/kg body weight	[181]
		Treating thrombocytopenia	[180]		
***Aldehydes***	Vanillin	Neuroprotective, anti-inflammatory, antifungal, antibacterial, antiviral, and anticancer Modulates the activities of antibiotics	[182]	May pose health risks by increasing the absorption of drugs with moderate oral bioavailability.	[183]
***Carboxylic acids***	Cinamic acid	Antioxidant, antimicrobial, anticancer, neuroprotective, anti-inflammatory	[184]	Reduced for antidiabetic activity. Compared to some derivatives, it has reduced toxicity or no dermatological toxicity.	[186,187]
		Lipid-lowering, antiobesity, antihyperglycemic, cardioprotective, and vasorelaxant	[185]		
***P. cerasus***					
***Phenols***	Gallic acid	Antioxidant	[188]	Compared to other polyphenols, it has a high absorption	[196]
		Anti-inflammatory, antiobesity	[189]		
		It can be used to manage several neurological diseases and disorders, such as Alzheimer’s disease, Parkinson’s disease, stroke, sedation, depression, psychosis, neuropathic pain, anxiety, and memory loss, as well as neuroinflammation.	[190]		
		Anti-HIV, antiulcer, UV protection	[191]		
		Anticarcinogenic	[192]		
		Antioxidant and antineoplastic	[193]		
		Antiviral, antimicrobial, antiallergic, anti-melanogenic, neuroprotective, anti-Alzheimer’s, antidiabetic, and antiobesity	[194]		
		It can be used to treat atherosclerotic cardiovascular disease, coronary artery disease, and cerebral ischemia	[195]		
***Flavonoids***	Phloridzin	Antigenotoxic, antioxidant, anti-inflammatory, and anticarcinogenic	[197]	Possible adverse effects on the musculoskeletal system in conditions of hyperglycemia.	[201]
		Antiaging	[198]		
		Antiarthritic effect	[199]		
		Antidiabetic, antihyperglycemic, antibacterial, cardioprotective, neuroprotective, hepatoprotective, immunomodulatory, and antiobesity	[200]		
	Galangin	Anticancer (breast, renal, lung, esophageal, laryngeal, ovarian, cervical, colon)	[202]	Very low oral bioavailability	[206]
		Strong ability to control apoptosis and inflammation	[203]		
		Antioxidant, anti-inflammatory, antiarthritic	[204]		
		Hepatoprotectors	[205]		
***Coumarin***	Scopoletin	Antioxidant	[207]	Low, but which can be positively influenced by encapsulation in Solupus micelles It does not present toxicity	[210,211]
		Antimicrobial, immunomodulatory, anti-inflammatory, anticarcinogenic, neuroprotective	[208]		
		Antibacterial, antifungal, antiparasitic, hepatoprotective, antihyperlipidemic, antidiabetic, antiangiogenesis, antihypertensive, analgesic, anti-immunomodropozic, antiallergic, antiaging, and antigout	[209]		
***Common bioactive compounds (Rubus and Prunus)***					
***Phenolic acids***	Caffeic acid	Anticancer (hepatocarcinoma)	[212]	Raised in free form. Some phenolic acids may present moderate toxicity, promoting irritation of the gastric mucosa, skin, or eyes	[229,230]
		Antidiabetic, antiobesity, antiarteriosclerotic, antidepressant, antibacterial, antiviral	[213]		
		Mild antiemetic properties	[214]		
		Antiproliferative, immunomodulatory and neuroprotective	[215]		
	*p*-Coumaric acid	Antioxidant, efficacy in hypopigmentation and depigmentation	[216]	Raised in free form. Some phenolic acids may present moderate toxicity, promoting irritation of the gastric mucosa, skin, or eyes	[229,230]
		Antioxidant, anticancer, antimicrobial, antiviral, anti-inflammatory, antiplatelet, anxiolytic, antipyretic, analgesic and antiarthritic	[217]		
	Ferulic acid	Dermato-protectors (UV, antipigmentation, regeneration) Used as a stabilizer in cosmetic products for vitamins C and E	[218]	Raised in free form. Some phenolic acids may present moderate toxicity, promoting irritation of the gastric mucosa, skin, or eyes	[229,230]
		Antioxidant, anti-inflammatory, antiangiogenic, antiallergic, antimicrobial, antiviral, neuroprotective, and anticancer	[219]		
		Pro-angiogenesis, antithrombosis, antiaging, analgesic, antithrombotic	[220]		
		Antidiabetic, cardioprotective, neuroprotective, and antiapoptotic	[221]		
		Hepatoprotectors	[222]		
	Chlorogenic acid	Antioxidant, antibacterial, hepatoprotective, cardioprotective, anti-inflammatory, antipyretic, neuroprotective, anti-obesity, antiviral, antimicrobial, antihypertensive, free radical scavenger, and central nervous system stimulant	[223]	Raised in free form. Some phenolic acids may present moderate toxicity, promoting irritation of the gastric mucosa, skin, or eyes	[229,230]
		Antidiabetic, antifibrotic, antimelanogenesis, antiallergic, antifungal, antiatherosclerosis, dermal protection	[224]		
	Ellagic acid	Effective in treating insomnia, fatigue, ischemia, colorectal cancer, and multiple sclerosis; improves physical endurance and lowers blood glucose levels	[225]	Raised in free form. Some phenolic acids may present moderate toxicity, promoting irritation of the gastric mucosa, skin, or eyes	[229,230]
		Antitumor, antioxidant, anti-inflammatory, antimutation, antiallergic	[226]		
		Role in treating/managing metabolic syndrome	[227]		
		Neuroprotectors, hepatoprotectors, cardioprotectors, antiphotoaging, depigmenting agent	[228]		
***Flavonols***	Quercetin	Anti-inflammatory, antiviral, antioxidant, and psychostimulant, immune-supporting properties	[102,103,104]	Low due to partial solubility in water, but also chemical stability It is not toxic to the human body at a consumption of 3–1000 mg/day.	[238,239]
		Anticancer effect against malignant gynecological tumors May improve hyperandrogenemia and insulin resistance	[231]		
		Antioxidant and neuroprotector for Alzheimer’s therapy	[232]		
		Antiatherosclerosis (increased absorption in association with lipids)	[233]		
		Liver and kidney protection	[234]		
		Antihypertensive, ability to protect low-density lipoprotein (LDL) oxidation, and ability to inhibit angiogenesis	[235]		
		Antifungal, antiasthmatic, antiallergic, antiobesity	[236]		
		Antiaging, antiviral (COVID-19)	[237]		
	Catechin	Antioxidant, anticancer, antiobesity, (in excess, it can promote hepatitis)	[240]	Intestinal absorption and bioavailability are increased by formulations with sucrose and ascorbic acid	[243]
		Antihypertensive, anticoagulant, antiulcer, antithyroid, antihyperlipidemic, antidiabetic, antiosteoporotic, antiosteopenic, hepatoprotective, nephroprotective, neuroprotective, antiallergic, anxiolytic, antimicrobial	[241]		
		It can chelate metals essential for bacterial growth, which supports bactericidal action.	[242]		
***Flavonoids***	Rutin	Sedative, diuretic, analgesic, anticonvulsant, anticancer, antidepressant, antiarthritic, antidiabetic, antiulcer, antiasthmatic, anticataract, antiosteoporotic, antiosteopenic, antimicrobial, antifungal, antiviral, larvicidal, antimalarial, effective in atopic dermatitis	[244]	Limited. The lethal dose in mice is between 1.49 and 1.51 g/kg	[87,246]
		Effective in combating premenstrual dysphoric disorder	[245]		
***Tannins***	Proantho-cyanidins (oligo- or polymers of monomeric flavan-3-ols)	Antioxidant, anticancer, antidiabetic, neuroprotective, and antimicrobial	[247]	Low in oral administration Only monomers and oligomeric procyanidins with a degree of polymerization of less than 4 are absorbed. There is no evidence of toxicity when administered orally or of potential mutagenic action at a consumption of 2% of the diet.	[247,249,250]
		Hypolipidemic, anti-inflammatory, metabolic, and intestinal flora regulation, DNA repair	[248]		

**Table 5:** Synergistic activity of some bioactive compounds.

Chemical Compound	Synergistic Activity	References
Quercetin + vitamin C	Antiviral	[252]
Ellagic acid + gallic acid + catechin	Antibacterial	[253]
*p*-Coumaric acid + chlorogenic acid	Antibacterial	[254]
Proanthocyanidins + vitamin C + vitamin E	Synergistic effect for skin whitening	[248]
Catechin + quercetin	Against alcoholic liver damage	[255]
Catechin + vitamin E	Antioxidant	[256]
Ferulic acid + *δ*-tocotrienol (a derivative of vitamin E)	Against prostate cancer	[220]
Kaempferol + apigenin	Cytotoxic efficacy for colon cancer	[257]
Quercetin + astragalin	Anti-inflammatory	[258]
Quercetin + gallic acid + caffeic acid Quercetin + gallic acid + rutin	Antioxidant	[259]
Apigenin + naringenin	Anticarcinogenic	[260]
Flavonoids + tocopherol	Inhibitors in the case of lipid peroxidation	[256]
Isorhamnetin + quercetin	Potentiation of anticancer activity and broadening of the spectrum	[261]
Isorhamnetin + caffeic acid	Increased antioxidant activity	[261]
Chrysin + apigenin	Antitumor	[262]
Kaempeferol + chrysin	Anti-inflammatory and antioxidant	[263]
Betaine-salicylic acid cocrystal	Acne treatment	[264]
Vanillin + norfloxacin (antibiotic)	Antibacterial	[265]
Juglanin + doxorubicin (antitumor drug)	High cytotoxicity	[266]
Chrysin + radiotherapy	Anticarcinogenic	[267]
Toxifolin + chemotherapy	Anticarcinogenic	[268]

## Data Availability

Not applicable.
